# A Novel Autophagy-Related lncRNA Gene Signature to Improve the Prognosis of Patients with Melanoma

**DOI:** 10.1155/2021/8848227

**Published:** 2021-06-18

**Authors:** Yi Ding, Tian Li, Min Li, Tuersong Tayier, MeiLin Zhang, Long Chen, ShuMei Feng

**Affiliations:** ^1^Department of Histology and Embryology, School of Basic Medical Sciences, Xinjiang Medical University, Urumqi, Xinjiang, China; ^2^Department of Pharmacology, Pharmacy College, Xinjiang Medical University, Urumqi, China; ^3^Xinjiang Urumqi City Center Blood Station, Urumqi, China

## Abstract

**Objective:**

Autophagy and long noncoding RNAs (lncRNAs) have been the focus of research on the pathogenesis of melanoma. However, the autophagy network of lncRNAs in melanoma has not been reported. The purpose of this study was to investigate the lncRNA prognostic markers related to melanoma autophagy and predict the prognosis of patients with melanoma.

**Methods:**

We downloaded RNA sequencing data and clinical information of melanoma from the Cancer Genome Atlas. The coexpression of autophagy-related genes (ARGs) and lncRNAs was analyzed. The risk model of autophagy-related lncRNAs was established by univariate and multivariate Cox regression analyses, and the best prognostic index was evaluated combined with clinical data. Finally, gene set enrichment analysis was performed on patients in the high- and low-risk groups.

**Results:**

According to the results of the univariate Cox analysis, only the overexpression of LINC00520 was associated with poor overall survival, unlike HLA-DQB1-AS1, USP30-AS1, AL645929, AL365361, LINC00324, and AC055822. The results of the multivariate Cox analysis showed that the overall survival of patients in the high-risk group was shorter than that recorded in the low-risk group (*p* < 0.001). Moreover, in the receiver operating characteristic curve of the risk model we constructed, the area under the curve (AUC) was 0.734, while the AUC of T and N was 0.707 and 0.658, respectively. The Gene Ontology was mainly enriched with the positive regulation of autophagy and the activation of the immune system. The results of the Kyoto Encyclopedia of Genes and Genomes enrichment were mostly related to autophagy, immunity, and melanin metabolism.

**Conclusion:**

The positive regulation of autophagy may slow the transition from low-risk patients to high-risk patients in melanoma. Furthermore, compared with clinical information, the autophagy-related lncRNA risk model may better predict the prognosis of patients with melanoma and provide new treatment ideas.

## 1. Introduction

Melanoma is a highly malignant tumor characterized by strong invasiveness and metastasis [1]. Although it accounts for a low proportion of skin cancer cases, it is associated with a high mortality rate compared with other skin cancers [2]. Its occurrence is mainly caused by long-term exposure to ultraviolet radiation, sunburn, physical stimulation, and other factors [3]. These risk factors can promote the malignant transformation of melanocytes. At present, the treatment of melanoma mainly involves surgery, radiotherapy, chemotherapy, and other means to kill tumor cells and promote tumor cell apoptosis [4–6]. However, the mechanism of melanoma is regulated by a complex molecular regulatory network; hence, the effectiveness of currently available treatments is limited. In addition, according to the literature, autophagy-related genes (ARGs) and TNM staging can predict the prognosis of patients with melanoma [7, 8]. However, due to the heterogeneity of tumors, TNM staging cannot accurately predict the prognosis of patients. Therefore, early diagnosis and treatment of patients with melanoma are particularly important.

Autophagy is a catabolic process capturing proteins or organelles that need to be degraded and maintaining cell stability through lysosomes [9]. At present, studies have shown that autophagy plays an important role in the formation of tumors [10]. In the early stages of cancer, autophagy can inhibit tumor growth [11]. However, with the development of cancer, cancer cells show a high degree of autophagy dependence, and autophagy plays a role in promoting the growth of such cells [12]. In addition, autophagy can be used as a prognostic marker for a variety of cancers, while inhibition or activation of autophagy can destroy tumor cells [13–15]. Therefore, autophagy also plays an important role in the diagnosis and treatment of cancer.

Long noncoding RNAs (lncRNAs) are noncoding protein RNAs, with a length > 200 bp. In recent years, lncRNAs have been identified as important factors in the regulation of cancer, autoimmune disease, cardiovascular disease, etc. [16]. They can affect gene expression by regulating biological processes, including the binding of transcription factors, regulation of chromatin structure, and induction of miRNA, and subsequently regulate the biological function of cancer cells [17]. Current studies have shown that lncRNAs account for a large proportion of melanoma genomes compared with genes that encode proteins [18]; however, the mechanism of melanoma regulation remains unclear. Moreover, lncRNAs can also be used as diagnostic and prognostic markers to predict the prognosis of patients with melanoma [19]. Therefore, the role of lncRNA in melanoma is self-evident.

At present, numerous studies have confirmed the role of autophagy in melanoma [20, 21]. The increase in autophagy promotes the progression of melanoma [21], but it can also inhibit its growth [22]. Moreover, these studies are based on ARGs to diagnose or predict the prognosis of patients with this disease. In addition, recent studies have found that lncRNAs can directly or indirectly regulate autophagy [23]. They can directly activate the protein that initiates autophagy or indirectly regulate the expression of ARGs through the competing endogenous RNA mechanism [24]. Therefore, the network between lncRNAs and ARGs may be the key to elucidating the autophagy mechanism of melanoma. However, there are few studies on autophagy-related lncRNAs in this setting, and fewer autophagy-related lncRNAs have been used to evaluate the prognosis of patients with melanoma. Therefore, it is important to clarify the autophagy mechanism of melanoma related to lncRNAs and identify new molecular therapeutic targets.

In this study, we downloaded melanoma expression data from the Cancer Genome Atlas (TCGA) and obtained seven lncRNAs related to autophagy. Further multivariate Cox regression analysis, construction of an autophagy-related coexpression network, and gene set enrichment analysis (GSEA) were performed. Most importantly, we constructed a risk model that can more accurately diagnose and predict the prognosis of patients than clinical information, suggesting that the autophagy-related lncRNA signature is a reliable predictor for patients with melanoma. The workflow of the specific analysis is shown in Supplementary Figure S1.

## 2. Materials and Methods

### 2.1. Data Preparation

We downloaded the FPKM data and related clinical information of 471 melanoma samples from TCGA (https://www.cancergenome.nih.gov/). Furthermore, the mRNA matrix and lncRNA matrix of melanoma were obtained. TCGA database is a public database containing 33 tumor samples and matching normal samples. At present, the research on ARGs is relatively perfect, and the Human Autophagy-dedicated Database (HADb) (http://www.autophagy.lu/) is the first public database dedicated to human autophagy [25]. Hence, we downloaded ARGs from the HADb.

### 2.2. Coexpression Analysis of Autophagy Genes

After obtaining the autophagy genes from the HADb website, we compared the mRNA matrix of melanoma with this gene set and further extracted the autophagy-related mRNA matrix of melanoma. Finally, the coexpression network of the mRNA matrix and lncRNA matrix related to melanoma autophagy was analyzed using the limma package of the R software (version 4.0.2). ∣Correlation coefficient | ≥5 and *p* value < 0.001 were the best screening criteria [26, 27].

### 2.3. Survival Analysis of lncRNAs Associated with Autophagy

Prior to the multivariate Cox analysis of lncRNAs, we evaluated the significance of lncRNAs in survival through single-factor Cox analysis and conducted a univariate Cox analysis of the subsequent lncRNAs. We combined the survival time and survival status of the clinical information with lncRNAs and subsequently performed the Kaplan–Meier survival analysis of the autophagy-related lncRNAs through the survival package of the R software. *p* values < 0.05 denoted statistically significant differences.

### 2.4. Cox Regression Model Analysis

We further screened lncRNAs that can be used for the prediction of prognosis of patients. We used the survival package of the R software to perform a stepwise Cox regression analysis of lncRNAs with survival significance and obtain a risk score. The patients were divided into high- and low-risk groups according to their risk score. At present, the combination of multiple genes has demonstrated potential for predicting the prognosis of patients. Thus, the lncRNAs were screened through survival analysis and receiver operating characteristic (ROC) curve analysis using the survivalROC package. Finally, based on the median value of the risk score, we drew the risk score, survival diagram, and heat map using the pheatmap package. To better predict the prognosis of patients, we used a survival package combined with clinical information of patients and the risk model constructed in this experiment for univariate and multivariate Cox analyses and obtained the related forest plots. Furthermore, the ROC curve related to clinical information was drawn to evaluate the prognostic value of the model. *p* value < 0.05.

### 2.5. Construction of a Coexpression Network

The Cytoscape software (version 3.7.2) was used to visualize the lncRNA-related autophagy coexpression networks [28]. The Sankey diagram can directly show the autophagy-related coexpression network of the high- and low-risk groups; hence, this diagram was constructed using the ggalluvial package of the R software. Hazard ratios (HR) > 1 and <1 indicated high and low risk, respectively.

### 2.6. GSEA

GSEA enables researchers to better understand the potential pathogenic mechanism in the high- and low-risk groups. Therefore, we analyzed the Gene Ontology (GO) and the Kyoto Encyclopedia of Genes and Genomes (KEGG) of the high- and low-risk groups through GESA (version 4.0.3) [29]. Normalized enrichment score ≥ 1, nominal *p* value ≤ 0.05, and false discovery rate *q* value ≤ 0.25 denoted statistical significance.

## 3. Results

### 3.1. Coexpression Analysis of Autophagy Genes

We downloaded a total of 232 ARGs from the HADb database. In addition, by screening the mRNA matrix of TCGA, we obtained the mRNA expression matrix related to melanoma autophagy. Furthermore, we set ∣correlation coefficient | ≥5 and *p* value < 0.001 as the cut-off standards and used the limma package of the R software to analyze the coexpression of the mRNA matrix and lncRNA matrix of melanoma. Finally, we screened 89 mRNAs and 100 lncRNAs in patients with melanoma (Supplementary Figure S1).

### 3.2. Survival Analysis of lncRNAs Associated with Autophagy

Prior to the multivariate Cox regression analysis, we performed a univariate Cox survival analysis on 100 lncRNAs. Finally, the Kaplan–Meier survival analysis showed that 39 lncRNAs were closely related to the prognosis of patients with melanoma. The high expression of only two lncRNAs was significantly correlated with shorter overall survival (OS) in patients with melanoma, whereas the remaining 37 lncRNAs did not show such a relationship.

### 3.3. Multivariate Cox Regression Model Analysis

**Figure 1:**
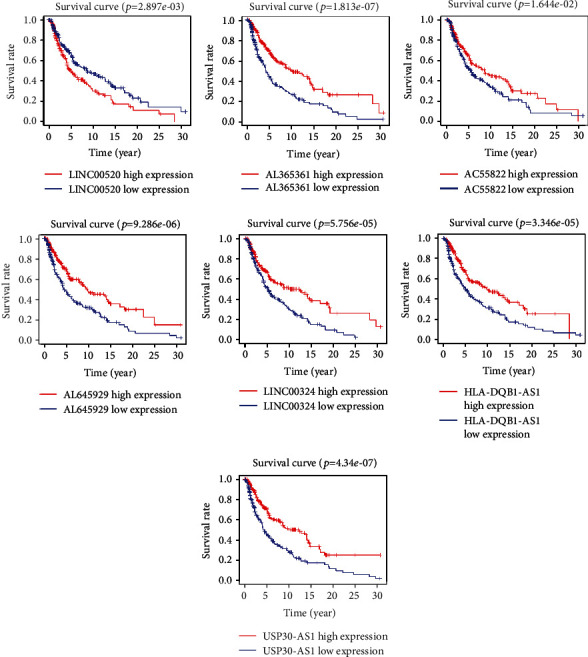
Survival analysis for the seven lncRNAs. lncRNA: long noncoding RNA.

**Figure 2:**
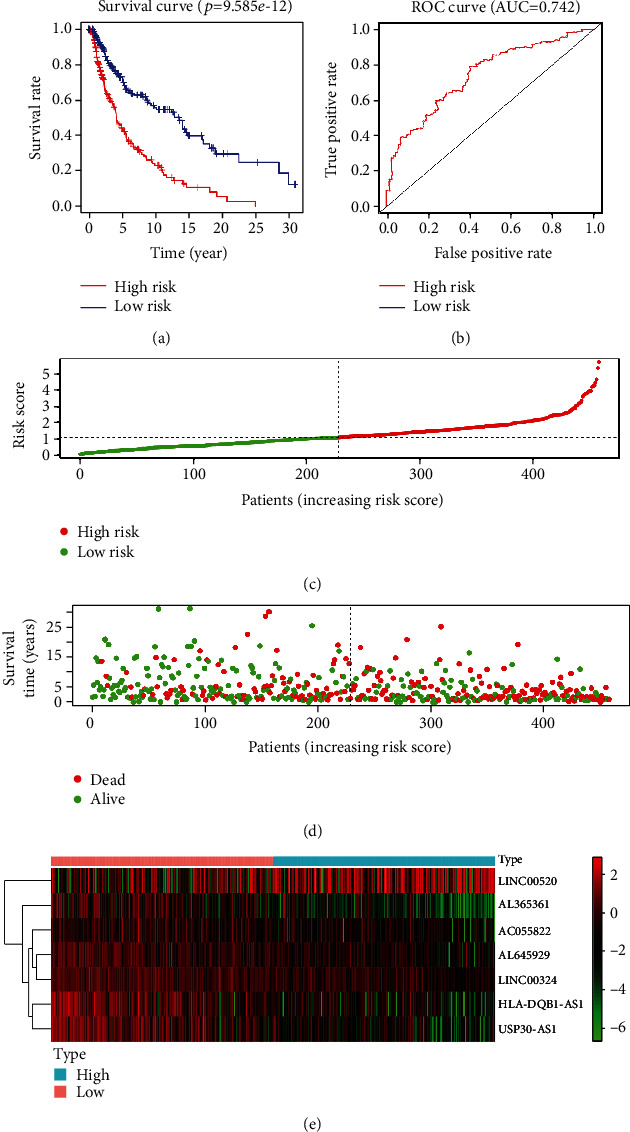
Multivariate Cox regression model analysis. (a) OS curves for the seven gene combinations in melanoma. (b) ROC curve and AUC. (c) Risk scores. Red indicates high risk. Green indicates low risk. (d) Survival diagram. Red nodes indicate death. Green indicates survival. (e) A heat map of seven genes. AUC: area under the curve; OS: overall survival; ROC: receiver operating characteristics.

The combination of multiple genes can better predict the prognosis of patients. Thus, we conducted a stepwise Cox regression analysis of 37 lncRNAs with survival significance and finally obtained a gene combination (Figure 1, Table 1) composed of seven lncRNAs (HLA-DQB1 antisense RNA 1 (HLA-DQB1-AS1), USP30 antisense RNA 1 (USP30-AS1), AL645929, AL365361, long intergenic nonprotein coding RNA 520 (LINC00520), LINC00324, and AC055822). In the multivariate Cox regression analysis, patients were divided into high- and low-risk groups according to the median value of their risk score. According to the risk score, we used the survivalROC package to draw the survival curve (Figure 2(a)) and ROC curve (Figure 2(b)) of this gene combination. Survival results showed that the high expression of this gene combination was significantly associated with poor OS in patients at high risk. The AUC of 0.742 suggests that this gene combination may be used to predict the prognosis of patients with melanoma. The risk score results showed that the high-risk group had a higher risk coefficient than the low-risk group, rising from left to right as illustrated in Figure 2(c). Compared with the low-risk group, the survival diagram results showed that the high-risk group was characterized by more deaths and shorter survival time (Figure 2(d)). Finally, the expression of seven genes in the high- and low-risk groups was visualized using a heat map (Figure 2(e)).

**Figure 3:**
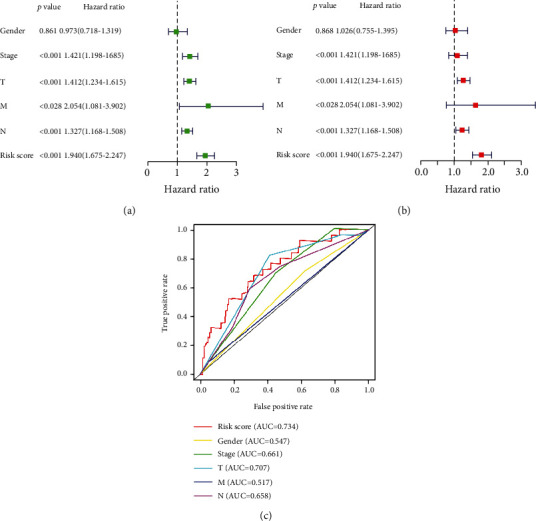
Independent prognostic analysis in melanoma: (a) forest plot of univariate Cox analysis; (b) forest plot of multivariate Cox analysis; (c) ROC curve and AUC. AUC: area under the curve; ROC: receiver operating characteristics.

In addition, to verify the higher accuracy of the risk model for predicting the prognosis of patients versus clinical information, we first performed a univariate Cox analysis based on clinical information, including sex, stage, T, M, and N. The forest plot results of the univariate analysis showed that stage, T, M, N, and risk score could be used to predict the prognosis of patients (Figure 3(a)). However, we used a multivariate Cox regression analysis and ROC curve to draw the clinical information and determine the index that may be used as the best independent prognostic factor. The forest plot results of the multivariate Cox analysis showed that T, N, and risk score could be used as independent prognostic factors (Figure 3(b)). However, the ROC results showed that the AUC of risk score, T, and N was 0.734, 0.707, and 0.658, respectively (Figure 3(c)). Therefore, the risk model we constructed is more accurate than T and N in predicting patient survival and is better than other traits (Table 2).

### 3.4. Construction of the Coexpression Network

**Figure 4:**
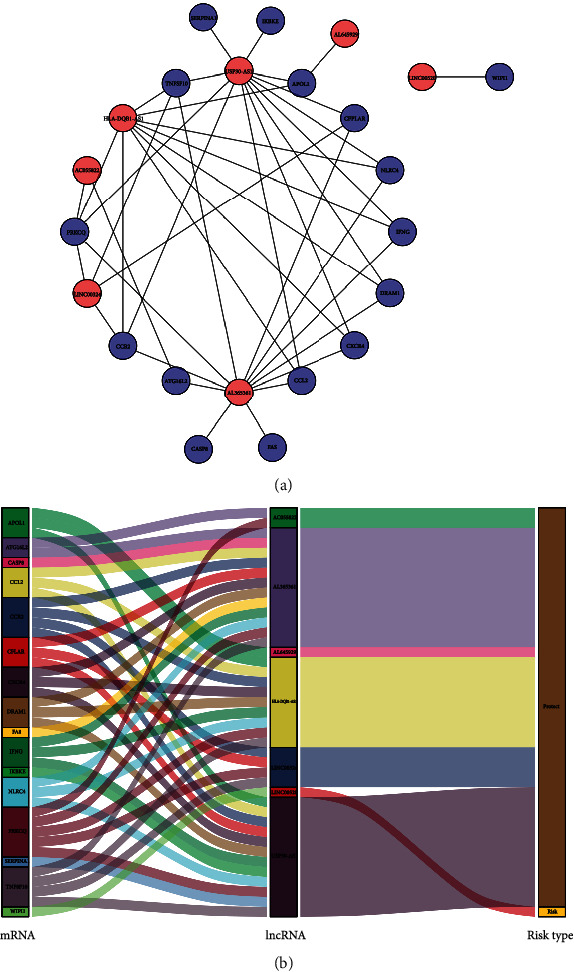
Coexpression network of autophagy. (a) Coexpression network constructed in Cytoscape. Red indicates lncRNAs. Blue indicates mRNAs. (b) Sankey diagram. lncRNA: long noncoding RNA; mRNA: messenger RNA.

We constructed an autophagy-related coexpression network of seven lncRNAs and 16 mRNAs using the Cytoscape software (Figure 4(a), Table 3). HR > 1 indicated a risk factor, whereas HR < 1 indicated a protective factor. We found that, in the coexpression network, only LINC00520 was a risk factor, whereas the other six lincRNAs were protective factors. Of note, the Sankey diagram yielded consistent results (Figure 4(b)).

### 3.5. GSEA in Melanoma

**Figure 5:**
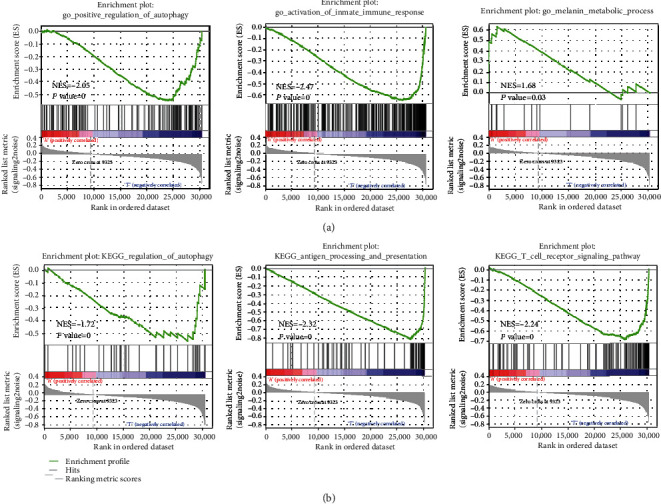
GSEA: (a) biological processes in melanoma; (b) KEGG analysis comparing the high- and low-risk groups. GSEA: gene set enrichment analysis; KEGG: Kyoto Encyclopedia of Genes and Genomes.

We used GSEA to analyze the GO and KEGG and study the potential pathogenic mechanism of patients at high and low risks of melanoma. The results of the GO enrichment analysis showed that the BP of the high-risk group was mainly related to the metabolism of melanin. The GO of patients at low risk was related to the positive regulation of autophagy and the activation of the immune response (Figure 5(a)). The results of KEGG were related to the regulation of autophagy, antigen processing and presentation, and T cell receptor signaling pathway (Figure 5(b)). Therefore, we hypothesized that the potential pathogenesis of melanoma in patients at high and low risks is related to the regulation of autophagy and the immune system. It was further demonstrated that the immune response plays an important role in the autophagy of melanoma.

## 4. Discussion

Melanoma is a disease associated with high mortality and difficult to diagnose in the early stage [30]. Therefore, there is an urgent need for the identification of molecular markers for early diagnosis and treatment. At present, studies have found that autophagy is closely related to the occurrence of melanoma [31]. However, most studies tend to evaluate the prognostic and therapeutic role of ARGs in melanoma. Thus far, there are no investigations on the diagnostic and prognostic value of autophagy-related lncRNA in melanoma. Hence, it is urgent to clarify the molecular mechanism of autophagy-related lncRNAs in melanoma and identify new molecular therapeutic targets.

In this study, we constructed an autophagy-related coexpression network composed of seven lncRNA and 16 mRNA genes. Following GSEA, we found that the GO in the low-risk group was more enriched in the activation of the immune system and the positive regulation of autophagy, while the enrichment results of KEGG were similar. Therefore, we hypothesized that autophagy and immune system regulation may affect the transition from low risk to high risk in melanoma. Univariate Cox regression showed that, among the seven examined lncRNAs, only LINC00520 was a risk factor, and its high expression was significantly correlated with poor OS in patients at high risk of melanoma. The remaining six lncRNAs were protective factors. Further multivariate Cox analysis showed that the gene set composed of the seven lncRNAs had high accuracy in predicting the prognosis of patients in the high- and low-risk groups.

According to the literature, the expression of mRNA and protein of autophagy genes decreased with the development of melanoma compared with the normal melanocyte [32]. Beclin 1 (BECN1) is a gene that activates autophagy and programmatically inhibits tumor proliferation. Light chain 3 (LC3) is closely related to the formation of the autophagy membrane. Miracco et al. found that the expression of these two autophagy genes decreased with the progression of melanoma compared with a benign nevus [33]. The expression of these two genes decreased with the increase in tumor grade and lymph node metastasis. A study conducted by Ding et al. on invasive liver cancer yielded similar findings [34]. Beclin 1 is an essential molecule for the formation of autophagosomes. It can mediate the localization of other autophagy proteins to autophagy vesicles to regulate the formation and maturation of autophagosomes. And it can comediate autophagy with the interaction of ARGS in some diseases reported in this paper. Jounai et al. found that NLRP4 can act as a negative regulator of autophagy and inhibit the occurrence of autophagy by binding to beclin 1 [35]. Besides, most studies judge the occurrence of autophagy by the expression level of beclin 1 and LC3. Cui et al. used beclin 1 and LC3 as indicators of autophagy and found that silencing DRAM led to the downregulation of beclin 1 and LC3 [36]. Similarly, He et al. found that TNFSF10 can promote the expression of beclin 1 as an inducing factor of autophagy [37]. Therefore, our follow-up mechanism study will take beclin 1/LC3 as the detection standard to further explore the relationship between autophagy-related lncRNA and ARGS. Also, bioinformatics analysis performed by Chen et al. revealed that the biological processes of the clear cell renal cell carcinoma (ccRCC) low-risk group were related to the regulation of autophagy [38]. Li et al. also found that the positive regulation of autophagy and enrichment of immune activation pathways were more associated with patients in the low-risk group than those in the high-risk group [14]. Moreover, in our study, activation of the immune system also occurred in patients in the low-risk group. In addition, autophagy can promote the development of T and B cells in cancer and be used to present antigens to major histocompatibility complex class II molecules, which can be recognized by the immune system to play an antitumor role. A good prognosis in the low-risk group may be related to autophagy and activation of the immune system. Therefore, the reduction of autophagy may increase the risk of melanoma.

The WD repeat domain phosphoinositide interacting 1 (WIPI1) is a member of the WIPI family [39]. It is involved in the formation of autophagosomes and the fusion of autophagy lysosomes. Studies have reported that WIPI1 was highly expressed in both prostate cancer and melanoma [40, 41]. Henry et al. pointed out that WIPI1 was highly expressed in melanoma cells compared with normal melanocytes, and tumor proliferation can be inhibited by inhibiting its expression [42]. In our study, WIPI1 and LINC00520 were coexpressed. As an oncogene, LINC00520 was highly expressed in numerous types of cancers. In breast cancer, LINC00520 promotes the proliferation of tumor cells by regulating STAT3 [42]. LINC00520 was also highly expressed in melanoma. Overexpression of LINC00520 can promote the proliferation, invasion, and migration of melanoma. At present, there is no report on LINC00520 and autophagy in any type of tumor [43]. Therefore, further experimental verification is warranted. In our autophagy coexpression network, LINC00324 was coexpressed with C-C motif chemokine receptor 2 (CCR2), CASP8, and FADD-like apoptosis regulator (CFLAR) and protein kinase C theta (PRKCQ). CCR2 is a specific chemokine for monocyte chemotaxis, playing a role in resisting many types of tumors in the human immune system [44]. According to the literature, CCR2 can enhance the monitoring of the immune system and play a role in tumor resistance by triggering the TH1 response and recruiting CD8+ cells [45]. CFLAR is an antiapoptotic protein, and its function is mostly related to the autophagy of T cells. In the body, a decrease in this autophagy gene can affect the development of peripheral T cells and decrease the proliferation ability of peripheral T cells [46]. Dunkle and He found that the rate of apoptosis of peripheral T cells with CFLAR deficiency increased [47]. Following bioinformatics analysis of ARGs in ccRCC, Miracco et al. found that PRKCQ was associated with poor OS in patients with ccRCC and may be used to evaluate their prognosis [33]. In summary, these genes are often associated with immune response and autophagy in tumors. According to our study, coexpression of CCR2, CFLAR, PRKCQ, and LINC00324 was associated with autophagy in melanoma. In our risk model, LINC00324 was a protective factor in patients at low risk. This observation may be related to activation of the immune response and positive regulation of autophagy in patients at low risk. Besides, in our study, HLA-DQB1-AS1, USP30-AS1, AL645929, AL365361, and AC055822 were also protective factors in patients at low risk. Moreover, in the coexpression network, they were closely related to CCR2, CFLAR, and PRKCQ. Interestingly, Sun et al. found that USP30-AS1 was significantly correlated with CFLAR, CXCR4, DRAM1, IFNG, IKBKE, and TNFSF10 in bladder cancer. And USP30-AS1 is also a protective factor in the bladder [48]. This is similar to what we found in melanoma. Therefore, USP30-AS1 may participate in the process of autophagy by affecting ARGs. Surprisingly, except for the high expression of LINC00520 in melanoma, thus far, we have not observed the expression and mechanism of the other six lncRNAs in melanoma. Moreover, we did not find any data reporting the role of these seven lncRNAs in the autophagy mechanism of melanoma. Therefore, our study provides a new idea for revealing the autophagy mechanism of this disease.

In recent years, a variety of functions of lncRNA have been continuously discovered, among which the study of the ceRNA mechanism is more in-depth. According to literature reports, some lncRNA can affect cell autophagy through the ceRNA mechanism and then lead to the occurrence of a variety of cancers and diseases. Liu et al. found that lncRNAGAS5 promotes autophagy of the colorectal cancer cell line (CRC) and inhibits the migration and invasion of CRC cells by regulating miR-222 and affecting the autophagy-related gene PTEN [49]. In colorectal cancer cells, lncRNA SNHG6 targets miR-26a-5p to promote chemotherapy through autophagy induced by ULK1 [50]. At the same time, the ceRNA network analysis related to autophagy in hepatocellular carcinoma was also verified by Yang et al. [51]. In this study, we found that autophagy-related lncRNA may affect the development of melanoma through ARGs. With the continuous increase in high-throughput sequencing and various databases, the mechanism of ceRNA has been further explored and verified: for example, LnCeVar and LncACTdb2.0 [52, 53]. These databases are more focused on predicting the ceRNA dataset of lncRNA and more accurately predicting the relationship between lncRNA and the disease. Therefore, these studies will improve our understanding of coding and noncoding RNA in complex diseases and promote our understanding of the relationship between autophagy and lncRNA from the perspective of ceRNA. Therefore, the autophagy-related lncRNA network we constructed may also be related to the ceRNA mechanism, but this still needs further research.

Autophagy was closely related to the prognosis of patients with melanoma. Hence, it is crucial to identify accurate prognostic markers for patients with melanoma. By constructing the coexpression network of ARGs and lncRNAs in melanoma, we performed univariate and multivariate Cox regression analyses of lncRNAs and eventually included seven lncRNAs in the risk model. To verify the specificity and accuracy of our risk model, we also performed univariate and multivariate Cox analyses using the clinical information of patients with melanoma. The final results showed that, compared with other clinical information, our risk model can more accurately predict the prognosis of patients with melanoma and provide new ideas for the mechanism and treatment of this disease.

## 5. Conclusion

We constructed an autophagy-related coexpression network using autophagy-related mRNAs and lncRNAs. This will provide a basis for our subsequent research on the autophagy mechanism of melanoma. In addition, we constructed a risk model, which may provide new insight into the diagnosis and prognosis of patients with melanoma in the future versus clinical information. Further investigations are warranted to verify the present findings.

## Figures and Tables

**Table 1:** Survival analysis for seven lncRNAs associated with autophagy. lncRNA: long noncoding RNA.

Gene	KM	*B*	SE	HR	HR.95L	HR.95H	*p* value
HLA-DQB1-AS1	3.35*E* − 05	-0.46	0.09	0.63	0.53	0.75	2.73*E* − 07
USP30-AS1	4.34*E* − 07	-0.50	0.09	0.61	0.51	0.72	2.22*E* − 08
AL365361	1.81*E* − 07	-0.49	0.11	0.62	0.50	0.76	1.11*E* − 05
LINC00520	2.90*E* − 03	0.13	0.04	1.13	1.06	1.22	4.50*E* − 04
LINC00324	5.76*E* − 05	-0.74	0.15	0.48	0.35	0.64	1.42*E* − 06
AC055822	1.64*E* − 02	-0.47	0.16	0.62	0.46	0.84	2.29*E* − 03

**Table 2:** Results of Cox analysis of the risk model and clinical data.

Cox	ID	*B*	SE	*z*	HR	HR.95L	HR.95H	*p* value
UniCox	Gender	-0.03	0.16	-0.18	0.97	0.72	1.32	8.61*E* − 01
	Stage	0.35	0.09	4.03	1.42	1.20	1.68	5.52*E* − 05
	T	0.34	0.07	5.02	1.41	1.23	1.62	5.20*E* − 07
	M	0.72	0.33	2.20	2.05	1.08	3.90	2.80*E* − 02
	N	0.28	0.07	4.34	1.33	1.17	1.51	1.39*E* − 05
	Risk score	0.66	0.07	8.84	1.94	1.67	2.25	9.74*E* − 19

MultiCox	Gender	0.03	0.16	0.17	1.03	0.76	1.39	8.68*E* − 01
	Stage	0.08	0.12	0.67	1.09	0.85	1.38	5.03*E* − 01
	T	0.24	0.07	3.37	1.27	1.11	1.46	7.46*E* − 04
	M	0.49	0.38	1.29	1.63	0.78	3.43	1.96*E* − 01
	N	0.21	0.08	2.66	1.23	1.06	1.43	7.78*E* − 03
	Risk score	0.59	0.08	7.55	1.81	1.55	2.11	4.21*E* − 14

**Table 3:** Coexpression network of autophagy.

ARGgene	lncRNA	Cor	*p* value
APOL1	HLA-DQB1-AS1	0.55	4.38*E* − 39
CCL2	HLA-DQB1-AS1	0.53	1.83*E* − 35
CCR2	HLA-DQB1-AS1	0.60	4.04*E* − 48
CXCR4	HLA-DQB1-AS1	0.53	2.62*E* − 35
DRAM1	HLA-DQB1-AS1	0.54	2.73*E* − 36
IFNG	HLA-DQB1-AS1	0.60	2.44*E* − 48
NLRC4	HLA-DQB1-AS1	0.55	5.89*E* − 39
PRKCQ	HLA-DQB1-AS1	0.58	2.77*E* − 44
TNFSF10	HLA-DQB1-AS1	0.60	7.52*E* − 48
APOL1	USP30-AS1	0.78	3.73*E* − 98
CCL2	USP30-AS1	0.58	8.08*E* − 43
CCR2	USP30-AS1	0.78	3.82*E* − 96
CFLAR	USP30-AS1	0.61	7.63*E* − 49
CXCR4	USP30-AS1	0.64	3.70*E* − 55
DRAM1	USP30-AS1	0.63	2.19*E* − 53
IFNG	USP30-AS1	0.86	6.41*E* − 142
IKBKE	USP30-AS1	0.54	2.51*E* − 36
NLRC4	USP30-AS1	0.66	5.48*E* − 61
PRKCQ	USP30-AS1	0.79	4.51*E* − 100
SERPINA1	USP30-AS1	0.68	6.25*E* − 66
TNFSF10	USP30-AS1	0.70	8.92*E* − 72
ATG16L2	AL365361	0.59	6.67*E* − 45
CASP8	AL365361	0.52	7.13*E* − 34
CCL2	AL365361	0.53	3.50*E* − 36
CCR2	AL365361	0.83	1.05*E* − 121
CFLAR	AL365361	0.68	2.52*E* − 65
CXCR4	AL365361	0.65	2.08*E* − 57
DRAM1	AL365361	0.53	4.36*E* − 36
FAS	AL365361	0.51	1.55*E* − 32
IFNG	AL365361	0.64	1.19*E* − 56
NLRC4	AL365361	0.67	1.03*E* − 62
PRKCQ	AL365361	0.77	2.36*E* − 95
TNFSF10	AL365361	0.66	1.33*E* − 59
CCR2	LINC00324	0.54	1.32*E* − 37
CFLAR	LINC00324	0.53	8.85*E* − 36
PRKCQ	LINC00324	0.52	2.84*E* − 34
TNFSF10	LINC00324	0.52	1.68*E* − 34
ATG16L2	AC055822	0.60	2.04*E* − 47
PRKCQ	AC055822	0.51	1.27*E* − 32
APOL1	AL645929	0.55	3.80*E* − 38
WIPI1	LINC00520	0.51	6.32*E* − 33

## Data Availability

All data analyzed during this study are included in this article.

## Supplementary Materials

Supplementary Materials Supplementary Figure S1: specific workflow.
