# Supplementary figures and images for: A Novel Autophagy-Related lncRNA Gene Signature to Improve the Prognosis of Patients with Melanoma
Source: Biomed Res Int. 2021 Jun 18;2021:8848227. doi: 10.1155/2021/8848227 (PMC8238568; doi:10.1155/2021/8848227)

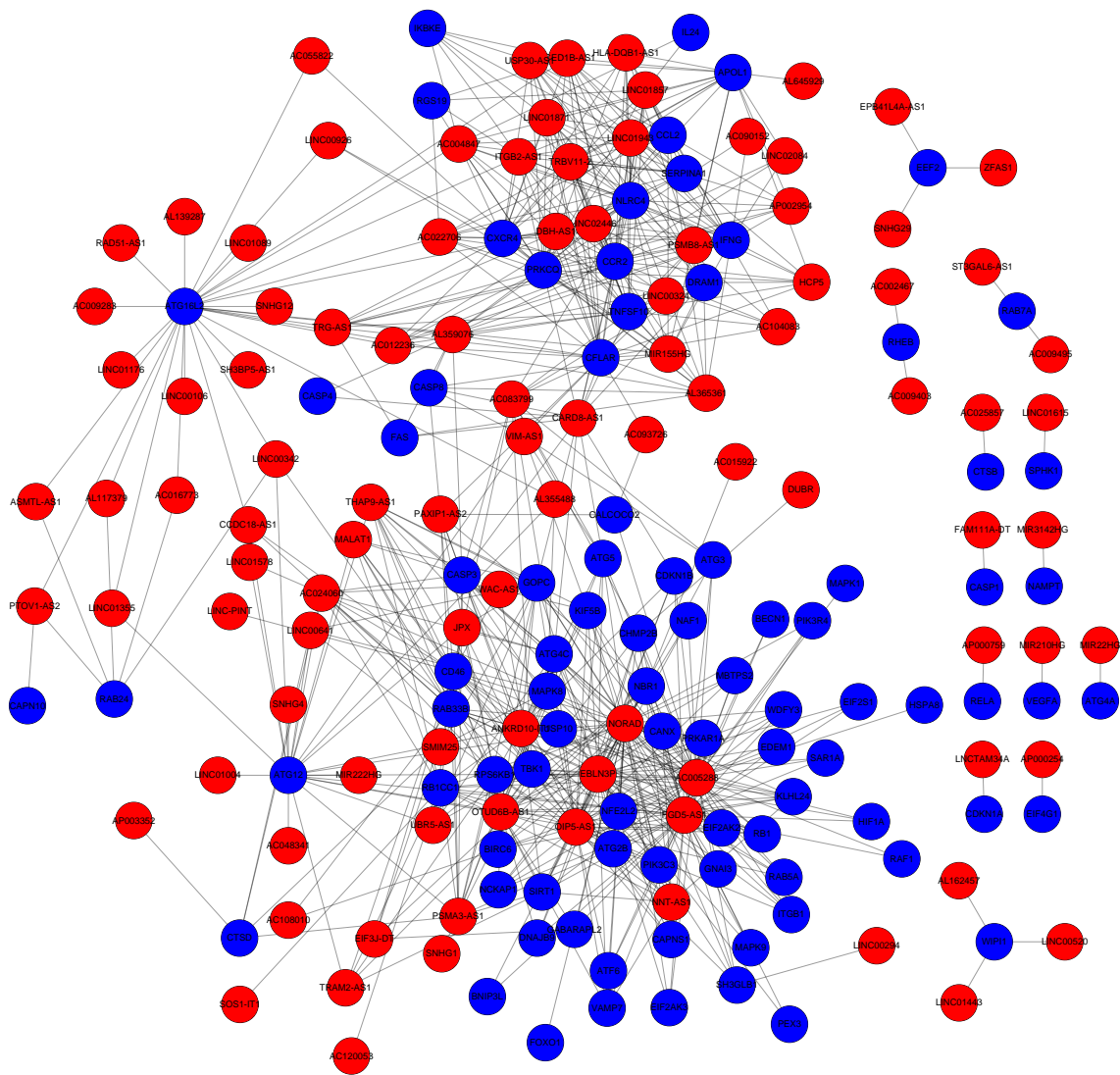

Supplement: Supplementary Materials — Supplementary Figure S1: specific workflow. [file 8848227.f1.pdf]
